# The NF-κB p65/miR-23a-27a-24 cluster is a target for leukemia treatment

**DOI:** 10.18632/oncotarget.5591

**Published:** 2015-09-10

**Authors:** Yong-Chang Zhang, Hui Ye, Zhi Zeng, Y. Eugene Chin, Yu-Ning Huang, Guo-Hui Fu

**Affiliations:** ^1^ Pathology Center, Shanghai General Hospital/Faculty of Basic Medicine, Shanghai Jiao Tong University School of Medicine, Shanghai, China; ^2^ Institute of Health Sciences, Shanghai Institutes for Biological Sciences (SIBS), Chinese Academy of Sciences (CAS) and Shanghai Jiao Tong University School of Medicine (SJTUSM), Shanghai, China

**Keywords:** NF-κB p65, miR-23a-27a-24 cluster, erythroid differentiation, leukemia

## Abstract

p65 is a transcription factor that is involved in many physiological and pathologic processes. Here we report that p65 strongly binds to the miR-23a-27a-24 cluster promoter to up-regulate its expression. As bone marrow-derived cells differentiate into red blood cells *in vitro*, p65/miR-23a-27a-24 cluster expression increases sharply and then declines before the appearance of red blood cells, suggesting that this cluster is negatively related to erythroid terminal differentiation. Bioinformatic and molecular biology experiments confirmed that the miR-23a-27a-24 cluster inhibited the expression of the erythroid proteome and contributed to erythroleukemia progression. In addition, high level of the p65/miR-23a-27a-24 cluster was found in APL and AML cell lines and in nucleated peripheral blood cells from leukemia patients. Furthermore, anti-leukemia drugs significantly inhibited the expression of the p65/miR-23a-27a-24 cluster in leukemia cells. Administration of the p65 inhibitor parthenolide significantly improved hematology and myelogram indices while prolonging the life span of erythroleukemia mice. Meanwhile, stable overexpression of these three miRNAs in mouse erythroleukemia cells enhanced cell malignancy. Our findings thus connect a novel regulation pathway of the p65/miR-23a-27a-24 cluster with the erythroid proteome and provide an applicable approach for treating leukemia.

## INTRODUCTION

Cell differentiation is a complex process that is tightly controlled by many cellular and molecular mechanisms [1, 2]. Differentiation of erythroid progenitors into red blood cells is one of the best characterized pathways [3–5]. Previous studies have elucidated the molecular events that occur during erythroid differentiation [6], and the role of transcription factors such as GATA-1, FOG-1 and Tal-1 that act by regulating the expression of a set of target genes [7–9]. Recent papers also reported that the transcription nuclear factor-κB p65 (NF-κB p65, referred to here as p65) is associated with erythroid differentiation [10–12].

NF-κB was initially discovered in B cells as a member of a family consisting of p65/RelA, p50, p52, RelB and c-Rel. All NF-κB members bind to κB sites in downstream gene promoters to regulate physiological and pathological processes including development, inflammation and immunity [13, 14]. NF-κB is generally sequestered in the cytoplasm and is maintained in an inactive form by the inhibitor IκB protein. Upon activation in response to several stimuli, NF-κB translocates to the nucleus to regulate gene expression [15]. Expression levels of the NF-κB subunits p65, p50, and p52 all change dramatically during early normal erythroid proliferation while NF-κB knockout mice show impaired erythropoiesis [16–18], which suggests that NF-κB factors modulate erythropoiesis. In addition, molecular evidence showed that NF-κB family members effectively repress globin gene expression [19]. Overexpression of p65 reduced the amounts of functional nuclear factor erythroid-derived 2 (NF-E2) proteins in K562 cells [11], suggesting that NF-κB expression is negatively correlated with erythroid differentiation. However, how p65 regulates its downstream target genes during erythroid differentiation and the roles of p65 in leukemia pathogens remain unclear.

In addition to transcription factors, microRNAs (miRNAs) have been shown to be important regulators of gene expression at the post-transcriptional level [20–22]. During erythropoiesis many miRNAs are induced or repressed [17, 23, 24], which suggests that miRNA-mediated regulation may guide the process of erythroid differentiation, although little is known about miRNA regulation and function during this process *in vivo*. In the present study, we show that p65 is a strong positive regulator of the miR-23a-27a-24 cluster. Moreover, these miRNAs target the erythroid proteome thus establishing a p65/miR-23a-27a-24/erythroid proteome pathway, the regulation of which plays an essential role in erythropoiesis. High expression level of the p65/miR-23a-27a-24 cluster is a vital pathogenesis factor of erythroleukemia and other types of leukemia.

## RESULTS

### p65 binds to the miR-23a-27a-24 cluster promoter and upregulates expression of the three miRNAs

**Figure 1:**
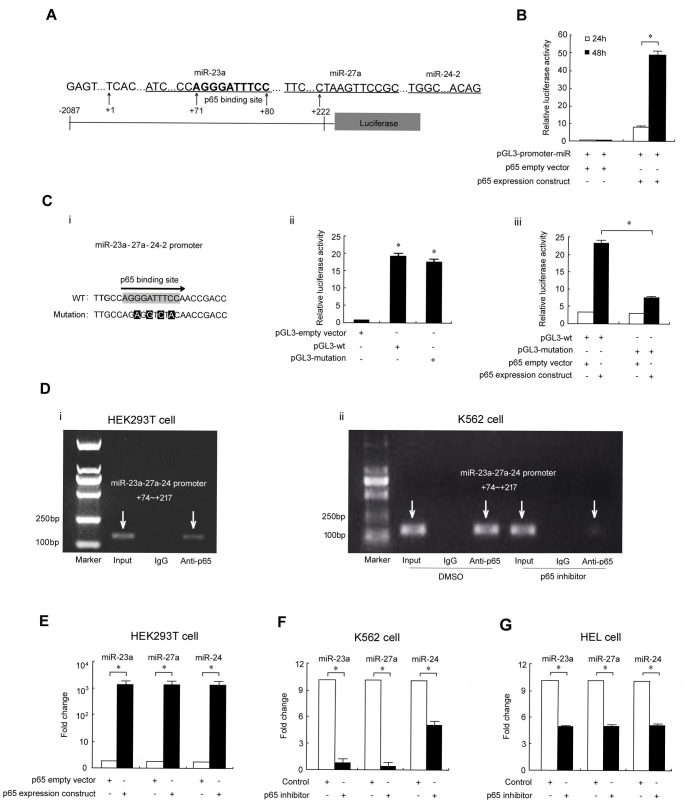
p65 binds to the miR-23a-27a-24 cluster promoter to upregulate expression **A.** Schematic diagram of promoter binding sites. Software TFSEARCH and TESS were used to predict the complete p65 binding site in the miRNA cluster promoter. **B.** Luciferase reporter gene assay. A Luc reporter vector spanning −2087 to +222 bases upstream of the three miRNAs was co-transfected into HEK293T cells with the p65 expression construct or empty vector. **C.** Point mutation of the p65 binding site in the miRNA cluster promoter region (i); effects of four point mutations on miRNA cluster promoter activity. *P < 0.01 compared with empty vector transfection (ii); effects of p65 overexpression on the activity of the miRNA cluster promoter with four point mutations. *P < 0.01 indicates a significant difference between WT (wild type) and mutant transfection (iii). **D.** ChIP assay revealed p65 binding to the miRNA cluster promoter in HEK293T (i) and K562 (ii) cells. **E.** Real-time PCR analysis of miRNA expression in HEK293T cells transfected with the p65 construct. *P < 0.01. **F.** Real-time PCR analysis of miRNA expression in K562 cells treated with p65 inhibitor parthenolide (10 μmol/ml, 48 h). **G.** Real-time PCR analysis of miRNA expression in HEL cells treated with p65 inhibitor parthenolide (2 μmol/ml, 72 h). *P < 0.05. Data are presented as mean±SD of three independent tests.

To explore the possible role of p65 in regulating the miR-23a-27a-24 cluster, we searched for potential p65 binding sites within the cluster promoter. As shown in Figure 1A, an AGGGATTTCC sequence that is a complete match to a typical p65 binding site was found in the +71~+80 region, which suggests that p65 might regulate expression of these three miRNAs. To test this possibility, a luciferase reporter vector spanning bases −2087 to +222 upstream of the three miRNAs was constructed and co-transfected with p65 expression constructs into HEK293T cells for 48 h. Overexpression of p65 strongly increased the luciferase activity in reporter vector transfected cells, with an approximately 50-fold increase in fluorescence as compared to cells transfected with the empty vector (Figures S1 and 1B). To confirm p65 binding to the promoter of the miRNAs, a mutation construct was generated and co-introduced with p65 expression constructs into HEK293T cells. Reporter assays demonstrated that a discontinuous mutation of four nucleic acids did not affect luciferase activity but significantly impaired the effect of p65 on the enzyme activity, which further indicated that p65 binds to this region (Figure 1C). We then examined p65 target occupancy in HEK293T and K562 cells by ChIP and found that p65 interacts with the upstream region of the miRNA cluster gene promoter (Figure 1D). Furthermore, p65 overexpression in HEK293T cells strongly up-regulated levels of the three miRNAs, with an approximately 1000-fold increase observed compared with the control transfection (Figure 1E). Meanwhile, treatment of the cells with the p65 inhibitor parthenolide significantly down-regulated the expression of these three miRNAs in K562 and HEL cells (Figure 1F and 1G).

### Dynamic changes of p65/miR-23a-27a-24 expression during erythropoiesis

**Figure 2:**
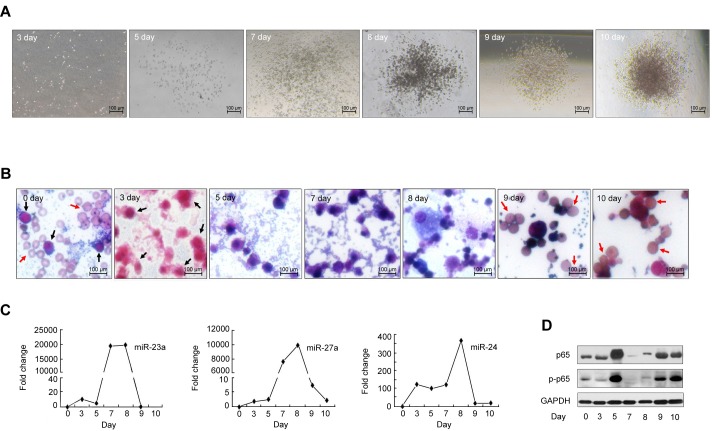
*In vitro* erythropoiesis BMDCs were isolated from the femurs and tibias of C57 mice and cultured in the special methylcellulose medium M3436 for 0, 3, 5, 7, 8, 9 and 10 days. The erythroid clones were observed and mature red blood cells identified by Wright's staining after the cells were cultured for 9-10 days (Figure 2A, 2B). **A.** Observation of erythroid colonies by microscopy at 5× magnification. **B.** Cell morphology with Wright's staining (20×). Black arrows indicate hemopoietic stem cells or hemopoietic progenitor cells. Red arrows indicate red blood cells. **C.** and **D.** MiRNAs, p65 and p-p65 expression was measured by real-time PCR and western blot at the above time points.

To determine whether the p65/miR-23a-27a-24 cluster is associated with erythroid differentiation, BMDCs were cultured in erythroid differentiation medium for 10 days using a protocol that was described previously [18, 25, 26]. The erythroid clones were progressively generated and mature red blood cells identified on day 9 and 10 of culture (Figure 2A and 2B). Under these culture conditions after 9 and 10 days of incubation 54% and 60%, respectively, of cells showed bare nuclei. Enucleating cells were readily detected throughout the culture during this time period. Parallel cell cultures were harvested at the same time points and used to measure miRNA and p65 protein. Induction of miRNAs began on day 5 and reached a maximum level by day 7 and 8 of culture (Figure 2C). Western blotting indicated that the amounts of total and phosphorylated (p-p65) were synchronously increased by day 5 of culture and declined by day 7 and 8 (Figure 2D). Cells with bare nuclei appeared after this reduction in miRNAs levels, suggesting that these three miRNAs are unfavorable factors for terminal erythroid differentiation and might be related to the development of erythroleukemia.

### High level of the p65/miR-23a-27a-24 cluster is a major event in erythroleukemia

**Figure 3:**
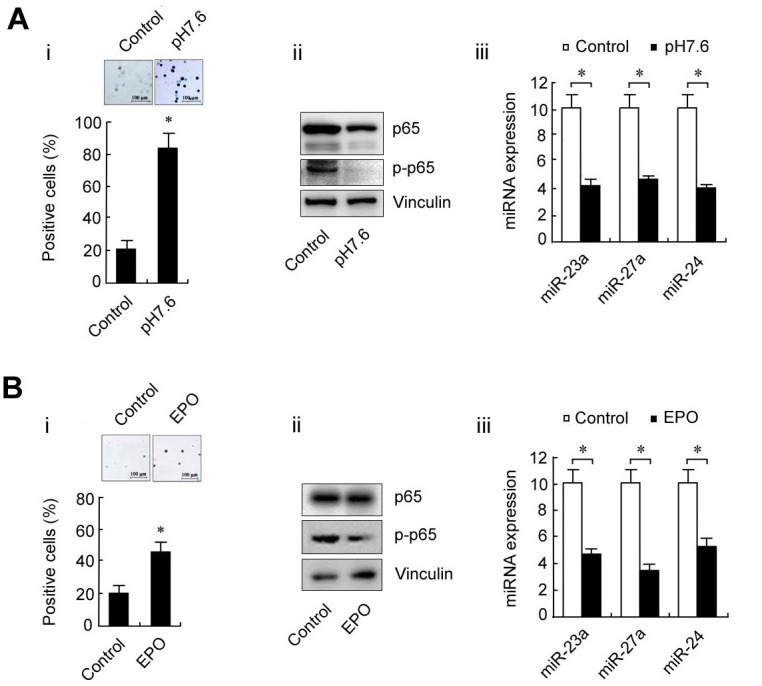
Changes in p65 and miRNAs during K562 cell differentiation **A.** K562 cells were cultured at pH7.6 for 6 days to induce erythroid differentiation. The differentiated cells were identified by benzidine staining and counted (i); p65 and p-p65 expression as detected by western blot (ii); miRNA levels as measured by real-time PCR (iii) before and after K562 cell differentiation. **B.** K562 cells were treated with EPO for 2 days to induce erythroid differentiation and the experiments were conducted as described in **A.**. Control was arbitrarily set as 10 (A, iii and B, iii). Data are presented as mean±SD of three independent tests. *P < 0.01. Images were taken with a Leica microscope at 10× magnification.

**Figure 4:**
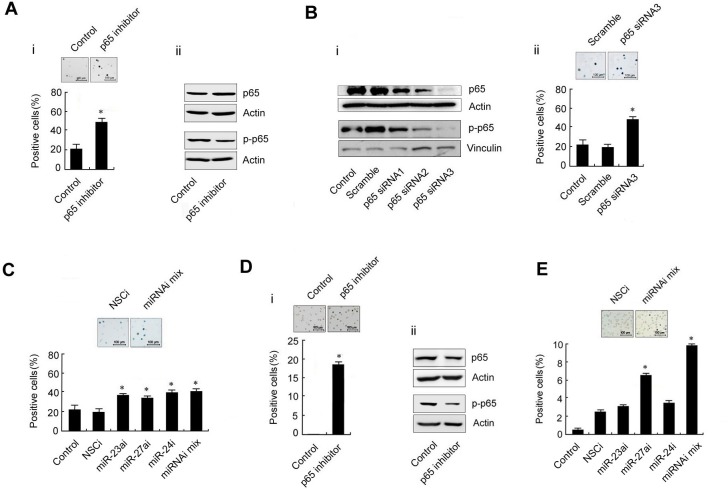
Differentiation of the human erythroleukemia cell lines K562 and HEL after treatment with a p65 inhibitor or transfection with p65-targeted siRNA or miRNA inhibitors **A.** K562 cells were treated with the p65 inhibitor parthenolide (10 μmol/ml, 48 h). The differentiated cells were detected by benzidine staining and positive cells were counted (i); the p65 and p-p65 expression was evaluated by western blot (ii). **B.** Expression of p65 and p-p65 in K562 cells transfected with p65-targeted siRNAs or scramble fragments for 48 h (i); K562 cells were transfected with siRNA3 or scramble fragments for 48 h and the cell differentiation was evaluated by benzidine staining (ii). **C.** The three miRNA inhibitors were transfected separately or together into K562 cells for 48 h as indicated and the differentiation of K562 cells was evaluated by benzidine staining. **D.** The experiments in **A.** were repeated in HEL cells. **E.** The experiments in **C.** were repeated in HEL cells. *P < 0.01 compared with control and non-specific control inhibitor (NSCi) transfection. Data are presented as mean±SD of three independent tests. *P < 0.05. Images were taken with a Leica microscope at 10× magnification.

To determine the role of the p65/miR-23a-27a-24 cluster in erythroleukemia progression, K562 cells were cultured in pH7.6 medium or treated with EPO (200 U/ml) for 48 h to induce differentiation and the differentiated cells were then evaluated by benzidine staining. Approximately 80% of K562 cells were differentiated at pH7.6, which is significantly higher than that of cells cultured under control conditions. Meanwhile, the expression of p65, p-p65 and three miRNAs were decreased along with the differentiation of K562 cells (Figure 3A). The same phenomenon also occurred following EPO-induced differentiation of K562 cells (Figure 3B). These results indicated that high levels of the p65/miR-23a-27a-24 cluster might be involved in the development of erythroleukemia. To further confirm that the high p65 activity is related to the arrest of K562 cell differentiation, the cells were treated with the p65 inhibitor parthenolide (10μmol/ml) for 48 h or transfected with p65-targeted siRNA for 48 h. Inactivation of p65 or down-regulation of p65 expression through parthenolide (Figure 4A) or siRNA (Figure 4B) indeed led to differentiation of K562 cells. In addition, K562 cells were transfected with three miRNA inhibitors or miRNA inhibitor mixture, respectively. As shown in Figure 4C, the miRNA inhibitors significantly induced differentiation of the cells. The results were confirmed in another human erythroleukemia cell line HEL (Figure 4D and 4E). These results indicated that high levels of the p65/miR-23a-27a-24 cluster contribute to the development of erythroleukemia.

### Expression of the p65/miR-23a-27a-24 cluster in other human leukemia cell lines and nucleated peripheral cells from leukemia patients

**Figure 5:**
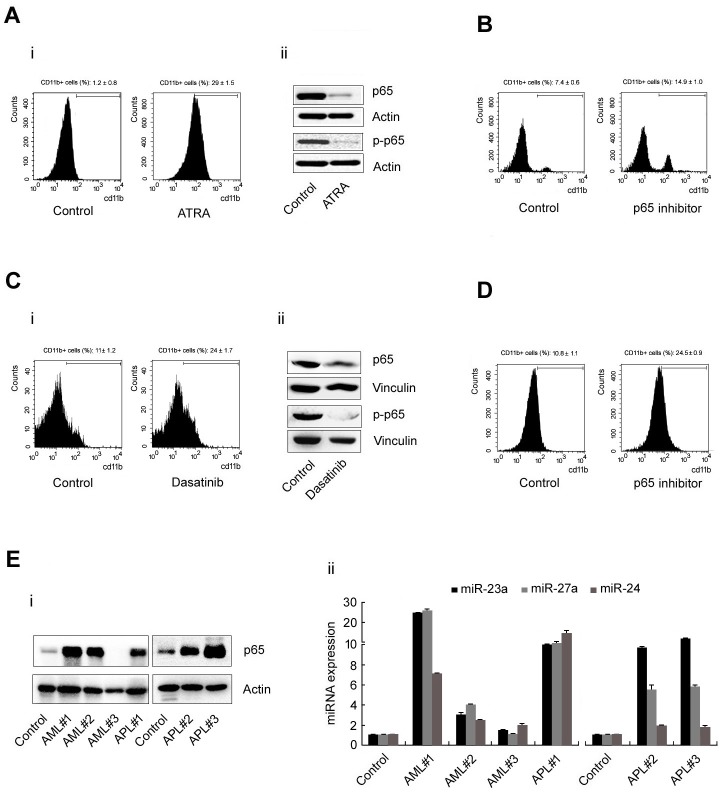
p65 and miRNA expression in leukemia cell lines and nucleated peripheral cells from leukemia patients **A.** NB4 cells were treated with ATRA (1mmol/L) for 3d and the cells were then harvested for several experiments: (i) CD11b+ cells counted by flow cytometry; (ii) p65 and p-p65 expression as detected by western blot. **B.** NB-4 cells were treated with p65 inhibitor for 3d and then the CD11b+ cells were counted by flow cytometry. **C.** Kasumi-1 cells were treated with dasatinib (100μmol/L) for 5d and experiments were performed as described in **A.**. **D.** Kasumi-1 cells were treated with p65 inhibitor for 3d and then the CD11b+ cells were counted by flow cytometry. **E.** The expression of p65 (i) and the expression of the miRNAs (ii) in nucleated peripheral cells from six leukemia patients were detected by western blot or real-time PCR. Data are presented as mean±SD of three independent tests. *P < 0.05.

To understand the expanding role of the p65/miR-23a-27a-24 cluster in leukemia progression, cells from the acute promyelocytic leukemia cell line (APL) NB4 were treated with all-trans-retinoic acid (ATRA, 1mmol/L) for 3d to induce differentiation. The CD11b^+^ cells were then counted by flow cytometry. Along with the differentiation of NB4 cells (Figure 5A, i), expression of p65, p-p65 was significantly decreased (Figure 5A, ii). Furthermore, treatment of NB4 cells with parthenolide (5μmol/ml) for 3d led to an increase in the CD11b^+^ cell population from 7.4% to 14.9% (Figure 5B). The same phenomenon also occurred when cells from the acute myelocytic leukemia (AML) cell line Kasumi-1 were treated with dasatinib (100μmol/L) for 5d. Approximately 24% of Kasumi-1 cells were CD11b^+^ in the dasatinib-treated group, while only 11% cells were CD11b^+^ in the control group (Figure 5C, i). Western blotting showed that p65 and p-p65 expression was decreased in the dasatinib-treated group (Figure 5C, ii). And treatment of Kasumi-1 cells with parthenolide (5μmol/ml) for 3 d led to an increase of CD11b^+^ cell population from 10.8% to 24.5% (Figure 5D). In addition, we collected six peripheral granulocyte samples from three AML patients and three APL patients and detected the level of the p65/miR-23a-27a-24 cluster in these samples. Both p65 (Figure 5E, i) and the three miRNAs (Figure 5E, ii) were highly expressed in all patient samples compared with samples from healthy subjects. These results indicated that the expression level of the p65/miR-23a-27a-24 cluster also participates in the progression of other types of leukemia.

### The p65/miR-23a-27a-24 cluster targets erythroid genes

**Figure 6:**
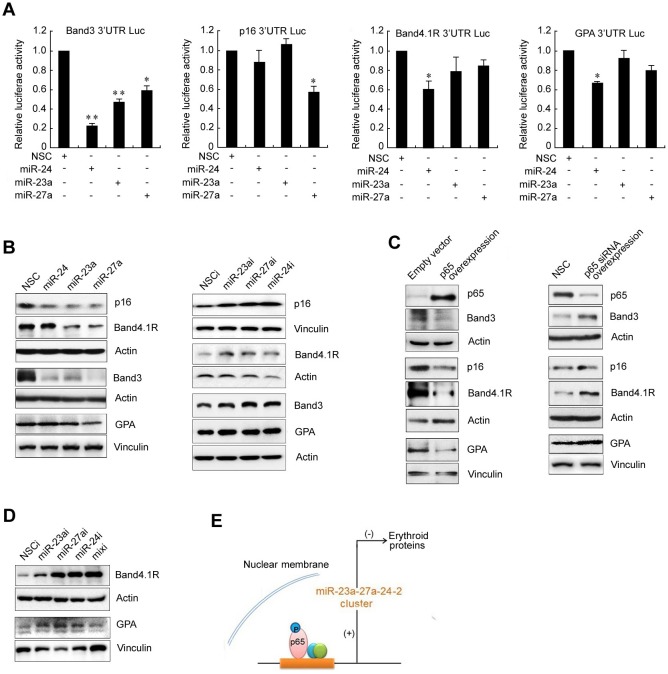
The miRNAs mediates p65-dependent suppression of band3, p16, band 4.1R and GPA expression **A.** Luc reporter gene assay. The 3′ UTRs of band3, p16, band4.1R and GPA genes were constructed into pGL3 Luc vectors and the constructs were co-transfected with miR-23a, miR27-a or miR-24 into HEK293T cells for 48 h. The Luc activities were then measured. *P < 0.05, **P < 0.01. **B.** MiRNA mimics (left) or inhibitors (right) were transfected into gastric cancer SGC7901 cells for 48 h and the expression of band3, p16,band4.1R and GPA was detected by western blot. **C.** The p65 expression construct or p65-targeted siRNA was transfected into SGC7901 cells for 48 h and band3, p16, band4.1R and GPA expression was detected by western blot. **D.** The miRNA inhibitors were transfected into K562 cells for 48 h and expression of band4.1R and GPA was detected by western blot. **E.** Schematic diagram of a novel signal pathway involving p65, the miRNA cluster and erythroid proteins.

We previously reported that miR-24 is involved in silencing the expression of band3 in K562 cells. Band3 is a major membrane protein that is specifically expressed on the surface of red blood cells. We therefore considered that the miR-23a-27a-24 cluster may target the functional proteome of red blood cells. To test this possibility, a bioinformatics method was used to analyze the potential effects of these three miRNAs on a set of functional proteins that are commonly recognized to play key roles during erythropoiesis. As shown in S4, all of the 3′ UTR or coding sequences of the analyzed erythroid genes carried a binding site for at least one miRNA, with a maximum of 17 binding sites in the IGF-1R gene. To test the inhibitory effects of the miR-23a-27a-24 cluster on erythroid gene expression, four typical erythroid genes band3, p16, GPA and band4.1R were selected for further investigation. The 3′ UTR of the four genes were cloned into a luciferase reporter vector and co-transfected with the miRNA mimics into HEK293T cells for 48 h. Luc experiment showed that the expression of each of these genes was affected by at least one of the three miRNAs (Figure 6A). To confirm that these three miRNAs can suppress the expression of band3, P16, GPA and band4.1R proteins, miRNA mimics or inhibitors were transfected for 48 h into gastric cancer SGC7901 cells, which express abundant erythroid proteins. Western blot showed that transfection of miRNA mimics significantly inhibited the expression of these proteins, while miRNA inhibitors increased their expression (Figure 6B). Furthermore, forced expression of p65 in SGC7901 cells down-regulated the expression of band3, P16, GPA and band4.1R proteins, with p65 knock-down up-regulating their expression (Figure 6C). Bioinformatics analysis showed that the entire promoter regions for band3, P16, GPA and band4.1R have no p65 binding sites (S2), and the effect of p65 target-siRNA on the expression of the erythroid protein was blocked by the miRNA mimics (S3). The results indicated that p65 inhibits the expression of erythroid proteins through the miR-23a-27a-24 cluster rather than directly through a gene promoter. Transfection of miRNA inhibitors into K562 cells also significantly increased the expression of band4.1R and GPA (K562 cells lack band3 and p16) (Figure 6D). Figure 6E shows a schematic diagram of the strong p65-dependent up-regulation of the miR-23a-27a-24 cluster and that the miR-23a-27a-24 cluster further inhibits erythroid protein expression (Figure 6E). Taken together, these results highlight a novel regulation pathway that links the p65/miR-23a-27a-24 cluster with erythroid protein expression to play a vital role in erythropoiesis. Dysregulation of this signal pathway may be closely related to the progression of leukemia.

### Application of a p65 inhibitor prolonged the lifespan of leukemia mice

**Figure 7:**
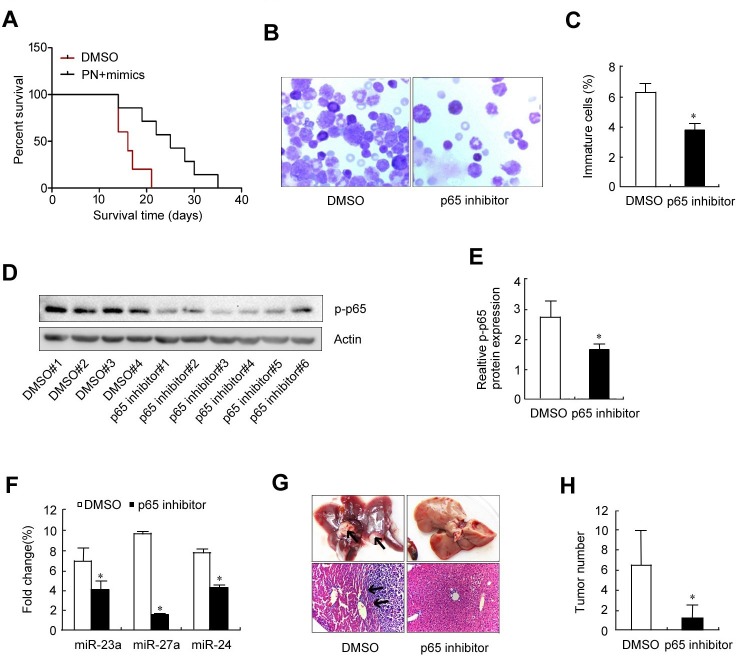
Therapeutic evaluation of the p65 inhibitor parthenolide on erythroleukemia mice Twelve C57 mice received tail vein injection of 2×10^6^ FBL-3 cells. The mice were treated (i.p.) with parthenolide (*n* = 7) or DMSO (*n* = 5) once every two days for three times and the therapeutic effects were evaluated. **A.** Mouse survival curves. **B.** Wright's staining of bone marrow smears. **C.** Cell count of immature bone marrow cells. **D.** Western blot analysis of p-p65 expression in bone marrow cells. **E.** Quantitative densitometric analysis of the western blot shown in **D.**; the data were normalized to the loading control, actin. *P < 0.01. **F.** Real-time PCR analysis of miRNAs expression in bone marrow cells. **G.** Macroscopic and microscopic appearances (H & E staining) of liver metastasis lesions. Black arrows in the upper left panel represent metastases; black arrows in the lower left panel represent the boundary between normal liver tissue and erythroleukemia cells. **H.** Metastasis count of liver lesion numbers. Data are presented as mean±SD of three independent tests. *P < 0.05. Images for B were taken with a Leica microscope at 20× magnification.

**Figure 8:**
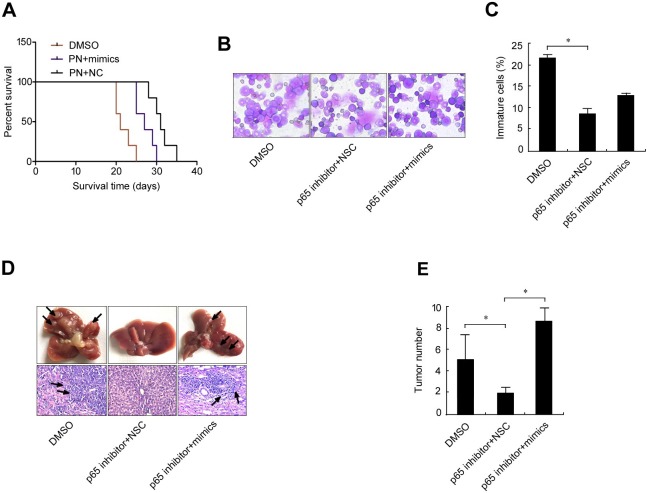
MiRNA mimics interfere the treatment effect of p65 inhibitor parthenolide on erythroleukemia mice Fifteen C57 mice received tail vein injection of 2×10^6^ FBL-3 cells. The mice were treated (i.p.) with parthenolide (*n* = 5) or parthenolide plus miRNA mimics (*n* = 5) or DMSO (*n* = 5) once every two days for three times. **A.**-**E.** The effects of the miRNA mimics on the role of p65 inhibitor were evaluated by the methods as described in Figure 7A-7C, 7G and 7H. Data are presented as mean±SD of three independent tests. *P < 0.05. Images for B were taken with a Leica microscope at 20× magnification.

If elevation of the p65/miR-23a-27a-24 cluster is a key event in leukemia, a p65 inhibitor could effectively relieve leukemia progression. To test this possibility, an erythroleukemia model was established in C57BL/6 mice by intravenously transplanting cells from the haploidentical mouse leukemic cell line FBL-3 at doses of 2×10^6^, which were established by previous studies. Seven experimental animals were administered with the p65 inhibitor parthenolide while five control animals were treated with DMSO. The control animals died on day 14, 14, 16, 17 and 21 after treatment, while the animals treated with the p65 inhibitor died on day 14, 19, 22, 25, 28, 30 and 35 after FBL-3 transplantation. Survival time analysis demonstrated that compared with control animals, the p65 inhibitor significantly prolonged the survival time of the erythroleukemia mice (Figure 7A). Examination of marrow smears from these mice showed that the number of immature cells was significantly lower in animals treated with the p65 inhibitor compared with control animals (Figure 7B and 7C). Meanwhile, p65 expression and that of the three miRNAs in bone marrow cells from the six animals treated with the p65 inhibitor was significantly down-regulated (Figure 7D–7F), which is similar to the findings from the *in vitro* evaluation in Figures 1 and 4. As soon as these mice died, the liver, spleen, lung and kidney were obtained for pathological examination, including assessment of p65 and miRNA expression levels as well as liver metastasis. The results showed that 100% incidences of erythroleukemia were observed in model mice. The survival time of the p65 inhibitor-treated mice was longer than the non-treated mice that were inoculated with the same number of tumor cells. Wright-Giemsa staining (Wright's staining) showed more mature cells in the bone marrow of p65 inhibitor-treated mice compared with the non-treated group. Liver tumor metastasis numbers were also counted, with p65 inhibitor-treated mice showing fewer metastases than the non-treated group (Figure 7G and 7H). In order to prove the existence of p65/miR-23a-27a-24 cluster axis in the control of erythroid differentiation, erythroleukemia mice were co-treated with p65 inhibitor and miRNA mimics. The results demonstrated that the miRNA mimics blocked the role of p65 inhibitor in prolonging the survival time of the erythroleukemia mice (Figure 8A), promoting the differentiation of leukemia cells (Figure 8B and 8C) as well as the suppression of liver metastasis (Figure 8D and 8E). Taken together, these results highlight the importance of the p65/miR-23a-27a-24 cluster in erythroleukemia progression.

### MiR-23a and miR-27a promote the progression of leukemia in mice

**Figure 9:**
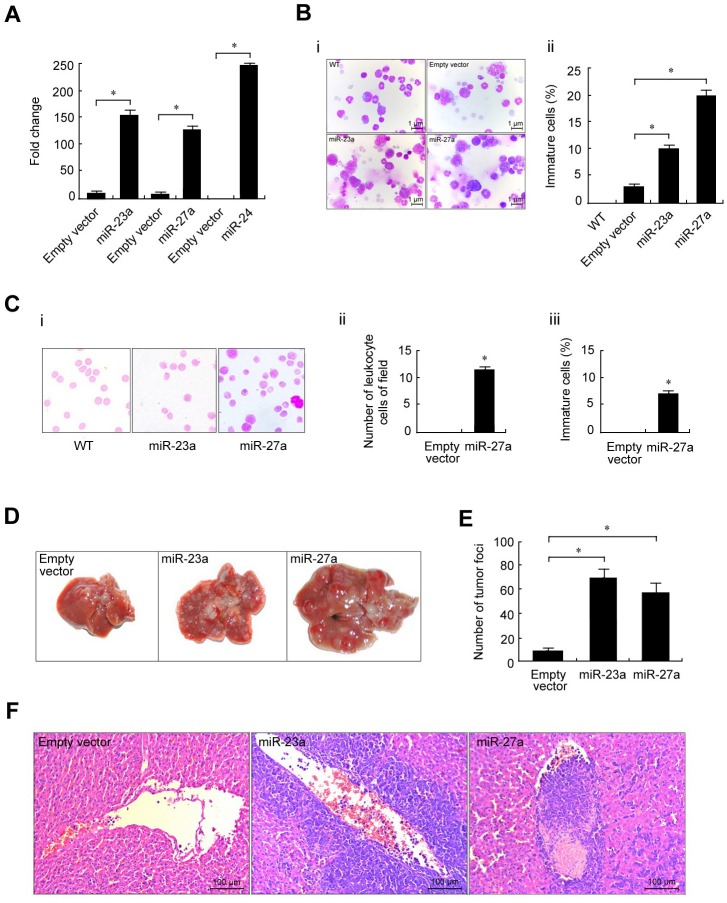
Stable overexpression of miR-23a, miR-27a and miR-24 promoted mouse erythroleukemia progression Recombinant pLVX-23a, pLVX-27a and pLVX-24 lentviruses used to infect FBL-3 cells are described in the Materials and Methods. **A.** The expression levels of miR-23a, miR-27a or miR-24 in FBL-3 cells were measured by real-time PCR. **B.** Lentivirus-infected FBL-3 cells were injected into mice through the tail vein. Wright's staining showed more immature cells in miRNA-overexpressing bone marrow compared with control animals that were injected with empty lentivirus vector (left, 40×) as indicated by the calculated percentage of immature cells (right). **C.** Left, peripheral blood smear by Wright's staining (100×oil); Right, immature and white blood cell counts of peripheral blood. **D.** Gross observation of metastatic lesions in the liver. Images were captured by NIKON D80 microscopy. **E.** The number of liver lesions. **F.** The microscopic appearance of liver lesions (H & E staining, 20×). Data are presented as mean±SD of three independent tests. *P < 0.05.

To further explore the role of the p65/miR-23a-27a-24 cluster in the progression of erythroleukemia, the three miRNAs were cloned into the lentivirus vector pLVX and stably transfected into FBL-3 cells. The cells overexpressing the three miRNAs were detected by real-time PCR and then intravenously injected into mice at doses of 2×10^6^ cells (*n* = 7) (Figure 9A). The bone marrow cells, liver, spleen, lung and kidney were obtained from dying C57BL/6 mice. Wright's staining of these tissues showed more immature cells in bone marrow from miRNA-overexpressing mice compared with control animals (Figure 9B). In addition, a large number of immature cells and leukocytes were observed in peripheral blood (Figure 9C). Meanwhile, more metastatic lesions in livers from miR-23a- and miR-27a-overexpressing mice were observed as compared with control mice (Figure 9D–9F).

## DISCUSSION

Transcription factors are essential for differentiation of specific blood lineages [27–30]. Unlike transcription factors, which are often absolutely required for specific genes, miRNAs may fine-tune gene expression by targeting a group of genes [31–33]. While changes in p65 levels during normal erythropoiesis and its activation in different types of leukemia have been reported, the erythroid differentiation mechanisms involving this protein and how they relate to leukemia progression are poorly understood. In the present paper, we provide evidence that a novel regulation pathway for erythropoiesis links p65 and the miR-23a-27a-24 cluster with erythroid protein expression. During erythroid differentiation, p65 and the three miRNAs first synchronously increased and then decreased. At the present time, we do not understand the reason and purpose of elevated expression of p65 and the miR-23a-27a-24 cluster in physiological erythropoiesis, but it is clear that expression levels of this protein and the miRNA cluster must decrease to ensure the induction of erythroid proteins, including band3, globin, band4.1R and GPA, which promote red blood cell maturation [34–36]. If such decreases in p65 and miR-23a-27a-24 cluster levels do not occur, expression of the erythroid proteins could be silenced and erythroid progenitor cell differentiation would be arrested, which in turn leads to malignant transformation. For example, band3 mRNA was detected in K562 cells but band3 protein expression was silenced. Transfection of a miR-24 inhibitor induced expression of band3 protein and promoted differentiation of K562 cells [37], suggesting that the miR-23a-27a-24 cluster can block erythroid terminal differentiation. To address why the p65/miR-23a-27a-24 cluster is maintained at very high levels in K562 cells, the 3′ UTR region of the p65 gene was cloned and sequenced, but no mutation was identified, suggesting that the p65/miR-23a-27a-24 is functional. Thus, whether this p65 overexpression is maintained by other mechanisms awaits further investigation. Nonetheless, these data demonstrated that sustained high levels of the p65/miR-23a-27a-24 cluster disturb normal hematopoiesis and contribute to leukemia progression.

The miR-23a-27a-24 cluster was found to have altered expression in several types of cancers with consistent or inconsistent expression [32]. In leukemia cells, this cluster was consistently found to be up-regulated in acute lymphoblastic leukemia, acute myeloid leukemia and chronic lymphocytic leukemia and down-regulated in acute promyelocytic leukemia [33, 38, 39]. In the present study we found that the miR-23a-27a-24 cluster is highly expressed in erythroleukemia K562 cells and plays a vital role in arresting cell differentiation.

Our findings provide a new strategy and target for the clinical treatment of leukemia. Both the p65 and miRNA inhibitors significantly and efficiently inhibited the malignancy of leukemia *in vitro* and *in vivo*, which strongly indicates that they could be potential drugs for treating leukemia. Our findings also connect a novel regulation pathway of the p65/miR-23a-27a-24 cluster with the erythroid proteome, which may also be applicable approach for designing therapies to target leukemia.

## MATERIALS AND METHODS

### Cells and media

Bone marrow-derived dendritic cells (BMDCs) were isolated from femurs and tibias of C57/BL mice treated for 4 days with 150 mg/kg/d 5-fluoro-uracil (5-FU). The BMDCs were cultured in serum-free methylcellulose medium (STEMCELL MethoCult™ SF M3436) for the Mouse Colony-Forming Cell Assay. All cell lines were cultured in RPMI-1640/Dulbecco's Modified Eagle's medium supplemented with 10% fetal bovine serum (20% for Kasumi-1), at 37°C and 5% CO_2_.

### Plasmid construction

Luciferase (Luc) reporter plasmids used in this study included the internal control vector pRL-TK, pGL3-basic vector, pGL3-promoter-miR, pGL3-3′UTR-band3, pGL3-3′ UTR-p16, pGL3-3′ UTR-band4.1R and pGL3-3′UTR-GPA. The pGL3-basic vector and internal control vector pRL-TK were purchased from Promega (Germany) and maintained in our lab. The pGL3-promoter-miR plasmid contained the miR-23a-27a-24 cluster promoter region. The pGL3-3′ UTR-band3, pGL3-3′ UTR-p16, pGL3-3′ UTR-band4.1R and pGL3-3′ UTR-GPA plasmids contain the 3′UTR of the band3, p16, band4.1R and glycophorinA (GPA) genes, respectively. The expression vectors pCMV4.0-empty and pCMV4.0-p65 were purchased from Addgene. The lentiviral vectors pMD2.G, psPAX2 and pLVX-IRES-ZsGreen1 were gifts from Professor Hong at the Shanghai Jiao Tong University School of Medicine. The pLVX-miR-23a, pLVX-miR-27a and pLVX-miR-24 lentiviral vectors contained miR-23a, miR-27a and miR-24, respectively.

### Antibodies and reagents

The anti-p65, anti-band3, anti-p16, anti-band4.1R, anti-GPA antibodies and anti-rabbit or anti-mouse IgG were purchased from Santa Cruz Biotechnology (Santa Cruz, CA, USA). The anti-phosphorylated p65 (p-p65) antibody was purchased from Cell Signaling Technology (Beverly, MA, USA). The p65 inhibitor parthenolide was purchased from Santa Cruz Biotechnology. The miRNA mimics, inhibitors, non-specific control (NSC), short interfering RNA (siRNA) smart pools (for p65) and scramble control were purchased from Pharma (Shanghai, China). All-trans-retinoic acid (ATRA) was purchased from Sigma. Dasatinib was a gift from the Shanghai Institute of Blood.

### Bioinformatics analysis

Prediction of miRNA targets was performed using TargetScan 5.1 and RNA22 software. Prediction of transcription factor binding sites was performed using TFSEARCH and TESS software.

### Luciferase assays

For miRNA target analysis, HEK293T cells were co-transfected with 600ng of the reporter vectors (pGL3-basic vector or various 3′UTR luciferase reporter vectors), 60ng of pRL-TK control vector and 30pmol of miRNA mimics. For functional analysis of miRNA cluster promoter activity, HEK293T cells were co-transfected with 600ng of pGL3-promoter-miR, 60ng of pRL-TK control vector and 600ng of pCMV.40-p65 expression construct or pCMV.40 empty vector. Cells were harvested 48 h post-transfection and assayed with a dual luciferase assay.

### Real-time quantitative PCR

Mature miRNA expression levels were detected by SYBR green real-time PCR. Data are expressed using the formula 2-ΔΔCT. For the miRNAs, U6 snRNA was used as the endogenous control. All PCR reactions were performed in triplicate.

### ChIP assay

Cells were incubated with 1% formaldehyde for 10 min, and then glycine was added to a final concentration of 0.125M to stop the cross-linking reaction. Cells were collected, washed, lysed in 500μl lysis buffer. Following 28 cycles of sonication at 4°C, the samples were centrifuged and the supernatant was diluted 4-fold with incubation buffer and subsequently mixed with antibodies or IgG for 2 h at 4°C. Then protein A sepharose beads were washed twice and pre-incubated with incubation buffer. The blocked protein A sepharose mixture was then combined with chromatin/antibody mixtures and incubated overnight. The beads were subsequently washed in a series of buffers. Protein-DNA complexes were eluted in elution buffer. To disrupt protein-DNA cross-links, eluted samples were supplemented and the DNA was purified and analyzed by RT-PCR using primers directed against a 144bp fragment spanning bases +74 to +217 of the miR-23a-27a-24 promoter.

### Western blot

For western blot analysis, cells were harvested. The samples were separated by SDS-PAGE, transferred to nitrocellulose (Millipore), and hybridized with antibodies. The immune complexes were detected by reaction with anti-rabbit or anti-mouse IgG conjugated to horseradish peroxidase, followed by ECL detection (Thermo).

### Oligonucleotides and transfection

MiRNA mimics, inhibitors and NSC were transfected with Lipofectamine 2000 reagent at a final concentration of 150pmol. SiRNA smart pools (for p65) and scramble control were transfected with Lipofectamine 2000 reagent at a final concentration of 100pmol. To obtain stably transfected FBL-3 cells, psPAX2 (a packaging plasmid), pMD2.G (an envelope plasmid) and pLVX-miR23a, pLVX-miR27a and pLVX-miR24 over-expression plasmids were separately co-transfected into HEK293T cells using X-treme GENE (Roche). After 48h, the harvested viral particles were used to infect FBL-3 cells with polybrene transfection, and centrifuged at 460×*g* for 2 h at 37°C. The infected cells were washed with 1640 medium and cultured for 48 h with GFP expression observed using fluorescence microscopy.

### Animal model

All C57BL/6 mice used in this study were bred and maintained in a pathogen-free environment. For the p65 inhibitor parthenolide treatment model C57BL/6 mice (6-8 weeks) were randomly divided into control (*n* = 5) and experimental (*n* = 7) groups. All mice received 2×10^6^ FBL-3 cells through intravenous lateral tail vein injection. After one week, the experimental mice were injected with 500μg/kg of parthenolide (i.p.). To evaluate the therapeutic effect of parthenolide on erythroleukemia mice, the survival time of mice was recorded and infiltration metastasis was assessed. For the miRNA overexpression model C57BL/6 mice (6-8 weeks) were randomly divided into FBL-3 control, pLVX-vector, pLVX-miR23a, pLVX-miR27a and pLVX-miR24 groups (*n* = 7 per group). The vector pLVX-miR23a, pLVX-miR27a and pLVX-miR24 group mice separately received 2×10^6^ pLVX-vector, pLVX-miR23a, pLVX-miR27a or pLVX-miR24 overexpressing FBL-3 cells through intravenous lateral tail vein injection. Infiltration metastasis was then assessed.

### Tissue processing

At necropsy haslets were removed and rinsed with PBS. For histopathology, tissues were fixed overnight in 10% neutral-buffered formalin, dehydrated, paraffin embedded and cut into 3 mm slices for hematoxylin and eosin (H&E) staining. Tissues were fixed overnight in 10% neutral-buffered formalin, dehydrated in 30% sucrose, routinely O.C.T. embedded and cut into 4 mm for frozen sections. GFP was observed directly by fluorescence microscopy.

### Patient samples

All patient samples were collected from Hunan Cancer Hospital, Hunan province, China. The human nucleated peripheral cells were isolated with lymphocyte separation medium (Corning, China), and expression of p65 and the three miRNAs was detected by Western blot or real-time PCR separately.

### Statistical analysis

Experimental data were analyzed with the SPSS statistical package version 13.0 (Chicago). All error bars represent the standard deviation (SD) of the mean. Differences between or among groups were tested using Student`s t-test or Mann-Whitney U test. Statistical differences were considered significant for *p* < 0.05 or *p* < 0.01.

## SUPPLEMENTARY MATERIAL FIGURES



## References

[1] Dustin P (1972). Cell differentiation and carcinogenesis: a critical review. Cell and tissue kinetics.

[2] Tsiftsoglou AS, Bonovolias ID, Tsiftsoglou SA (2009). Multilevel targeting of hematopoietic stem cell self-renewal, differentiation and apoptosis for leukemia therapy. Pharmacology & therapeutics.

[3] Kassouf MT, Chagraoui H, Vyas P, Porcher C (2008). Differential use of SCL/TAL-1 DNA-binding domain in developmental hematopoiesis. Blood.

[4] Cantu C, Ierardi R, Alborelli I, Fugazza C, Cassinelli L, Piconese S, Bose F, Ottolenghi S, Ferrari G, Ronchi A (2011). Sox6 enhances erythroid differentiation in human erythroid progenitors. Blood.

[5] Hattangadi SM, Wong P, Zhang L, Flygare J, Lodish HF (2011). From stem cell to red cell: regulation of erythropoiesis at multiple levels by multiple proteins, RNAs, and chromatin modifications. Blood.

[6] Woo AJ, Kim J, Xu J, Huang H, Cantor AB (2011). Role of ZBP-89 in human globin gene regulation and erythroid differentiation. Blood.

[7] Amigo JD, Ackermann GE, Cope JJ, Yu M, Cooney JD, Ma D, Langer NB, Shafizadeh E, Shaw GC, Horsely W, Trede NS, Davidson AJ, Barut BA, Zhou Y, Wojiski SA, Traver D (2009). The role and regulation of friend of GATA-1 (FOG-1) during blood development in the zebrafish. Blood.

[8] Mouthon MA, Bernard O, Mitjavila MT, Romeo PH, Vainchenker W, Mathieu-Mahul D (1993). Expression of tal-1 and GATA-binding proteins during human hematopoiesis. Blood.

[9] Rodriguez P, Bonte E, Krijgsveld J, Kolodziej KE, Guyot B, Heck AJ, Vyas P, de Boer E, Grosveld F, Strouboulis J (2005). GATA-1 forms distinct activating and repressive complexes in erythroid cells. The EMBO journal.

[10] Liu JJ, Hou SC, Shen CK (2003). Erythroid gene suppression by NF-kappa B. The Journal of biological chemistry.

[11] Lee WH, Chung MH, Tsai YH, Chang JL, Huang HM (2014). Interferon-gamma suppresses activin A/NF-E2 induction of erythroid gene expression through the NF-kappaB/c-Jun pathway. American journal of physiology Cell physiology.

[12] Li CY, Zhan YQ, Xu CW, Xu WX, Wang SY, Lv J, Zhou Y, Yue PB, Chen B, Yang XM (2004). EDAG regulates the proliferation and differentiation of hematopoietic cells and resists cell apoptosis through the activation of nuclear factor-kappa B. Cell death and differentiation.

[13] Kopp EB, Ghosh S (1995). NF-kappa B and rel proteins in innate immunity. Advances in immunology.

[14] Nairz M, Schroll A, Moschen AR, Sonnweber T, Theurl M, Theurl I, Taub N, Jamnig C, Neurauter D, Huber LA, Tilg H, Moser PL, Weiss G (2011). Erythropoietin contrastingly affects bacterial infection and experimental colitis by inhibiting nuclear factor-kappaB-inducible immune pathways. Immunity.

[15] Lernbecher T, Muller U, Wirth T (1993). Distinct NF-kappa B/Rel transcription factors are responsible for tissue-specific and inducible gene activation. Nature.

[16] Weih F, Durham SK, Barton DS, Sha WC, Baltimore D, Bravo R (1997). p50-NF-kappaB complexes partially compensate for the absence of RelB: severely increased pathology in p50(−/−)relB(−/−) double-knockout mice. The Journal of experimental medicine.

[17] Ma Y, Wang B, Jiang F, Wang D, Liu H, Yan Y, Dong H, Wang F, Gong B, Zhu Y, Dong L, Yin H, Zhang Z, Zhao H, Wu Z, Zhang J (2013). A feedback loop consisting of microRNA 23a/27a and the beta-like globin suppressors KLF3 and SP1 regulates globin gene expression. Molecular and cellular biology.

[18] Zhang MY, Sun SC, Bell L, Miller BA (1998). NF-kappaB transcription factors are involved in normal erythropoiesis. Blood.

[19] Wang Z, Liebhaber SA (1999). A 3′-flanking NF-kappaB site mediates developmental silencing of the human zeta-globin gene. The EMBO journal.

[20] Wang Q, Huang Z, Xue H, Jin C, Ju XL, Han JD, Chen YG (2008). MicroRNA miR-24 inhibits erythropoiesis by targeting activin type I receptor ALK4. Blood.

[21] Shen K, Mao R, Ma L, Li Y, Qiu Y, Cui D, Le V, Yin P, Ni L, Liu J (2014). A Post-Transcriptional Regulation of Tumor Suppressor MiR-139-5p and A Network of MiR-139-5p Mediated mRNAs Interactions in Colorectal Cancer. The FEBS journal.

[22] Filipowicz W, Bhattacharyya SN, Sonenberg N (2008). Mechanisms of post-transcriptional regulation by microRNAs: are the answers in sight?. Nature reviews Genetics.

[23] Li Y, Bai H, Zhang Z, Li W, Dong L, Wei X, Ma Y, Zhang J, Yu J, Sun G, Wang F (2014). The up-regulation of miR-199b-5p in erythroid differentiation is associated with GATA-1 and NF-E2. Molecules and cells.

[24] Lawrie CH (2010). microRNA expression in erythropoiesis and erythroid disorders. British journal of haematology.

[25] Wickrema A, Krantz SB, Winkelmann JC, Bondurant MC (1992). Differentiation and erythropoietin receptor gene expression in human erythroid progenitor cells. Blood.

[26] Huyhn A, Dommergues M, Izac B, Croisille L, Katz A, Vainchenker W, Coulombel L (1995). Characterization of hematopoietic progenitors from human yolk sacs and embryos. Blood.

[27] An X, Schulz VP, Li J, Wu K, Liu J, Xue F, Hu J, Mohandas N, Gallagher PG (2014). Global transcriptome analyses of human and murine terminal erythroid differentiation. Blood.

[28] Prasad P, Ronnerblad M, Arner E, Itoh M, Kawaji H, Lassmann T, Daub CO, Forrest AR, Lennartsson A, Ekwall K (2014). High-throughput transcription profiling identifies putative epigenetic regulators of hematopoiesis. Blood.

[29] Xu W, Carr T, Ramirez K, McGregor S, Sigvardsson M, Kee BL (2013). E2A transcription factors limit expression of Gata3 to facilitate T lymphocyte lineage commitment. Blood.

[30] Zhang Y, Lei CQ, Hu YH, Xia T, Li M, Zhong B, Shu HB (2014). Kruppel-like factor 6 is a co-activator of NF-kappaB that mediates p65-dependent transcription of selected downstream genes. The Journal of biological chemistry.

[31] Li S, Liu L, Zhuang X, Yu Y, Liu X, Cui X, Ji L, Pan Z, Cao X, Mo B, Zhang F, Raikhel N, Jiang L, Chen X (2013). MicroRNAs inhibit the translation of target mRNAs on the endoplasmic reticulum in Arabidopsis. Cell.

[32] Chhabra R, Dubey R, Saini N (2010). Cooperative and individualistic functions of the microRNAs in the miR-23a~27a~24-2 cluster and its implication in human diseases. Molecular cancer.

[33] Mi S, Lu J, Sun M, Li Z, Zhang H, Neilly MB, Wang Y, Qian Z, Jin J, Zhang Y, Bohlander SK, Le Beau MM, Larson RA, Golub TR, Rowley JD, Chen J (2007). MicroRNA expression signatures accurately discriminate acute lymphoblastic leukemia from acute myeloid leukemia. Proceedings of the National Academy of Sciences of the United States of America.

[34] Williamson RC, Toye AM (2008). Glycophorin A: Band 3 aid. Blood cells, molecules & diseases.

[35] Kaul RK, Kohler H (1983). Interaction of hemoglobin with band 3: a review. Klinische Wochenschrift.

[36] Nunomura W, Takakuwa Y, Cherr GN, Murata K (2007). Characterization of protein 4. 1R in erythrocytes of zebrafish (Danio rerio): unique binding properties with transmembrane proteins and calmodulin. Comparative biochemistry and physiology Part B, Biochemistry & molecular biology.

[37] Wu J, Zhang YC, Suo WH, Liu XB, Shen WW, Tian H, Fu GH (2010). Induction of anion exchanger-1 translation and its opposite roles in the carcinogenesis of gastric cancer cells and differentiation of K562 cells. Oncogene.

[38] Fulci V, Chiaretti S, Goldoni M, Azzalin G, Carucci N, Tavolaro S, Castellano L, Magrelli A, Citarella F, Messina M, Maggio R, Peragine N, Santangelo S, Mauro FR, Landgraf P, Tuschl T (2007). Quantitative technologies establish a novel microRNA profile of chronic lymphocytic leukemia. Blood.

[39] Saumet A, Vetter G, Bouttier M, Portales-Casamar E, Wasserman WW, Maurin T, Mari B, Barbry P, Vallar L, Friederich E, Arar K, Cassinat B, Chomienne C, Lecellier CH (2009). Transcriptional repression of microRNA genes by PML-RARA increases expression of key cancer proteins in acute promyelocytic leukemia. Blood.

