# Supplementary material for: The NF-κB p65/miR-23a-27a-24 cluster is a target for leukemia treatment
Source: Oncotarget. 2015 Sep 10;6(32):33554–67. doi: 10.18632/oncotarget.5591 (PMC4741785; doi:10.18632/oncotarget.5591)
Supplement: Supplementary file 1 [file oncotarget-06-33554-s001.pdf]

The NF-κB p65/miR-23a-27a-24 cluster is a target for leukemia treatment

Supplementary Material

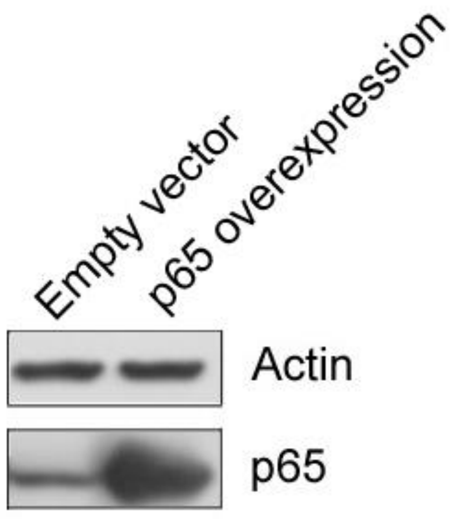

S1: Western blot analysis of p65 expression in HEK293T cells transfected with the p65 construct or empty vector.

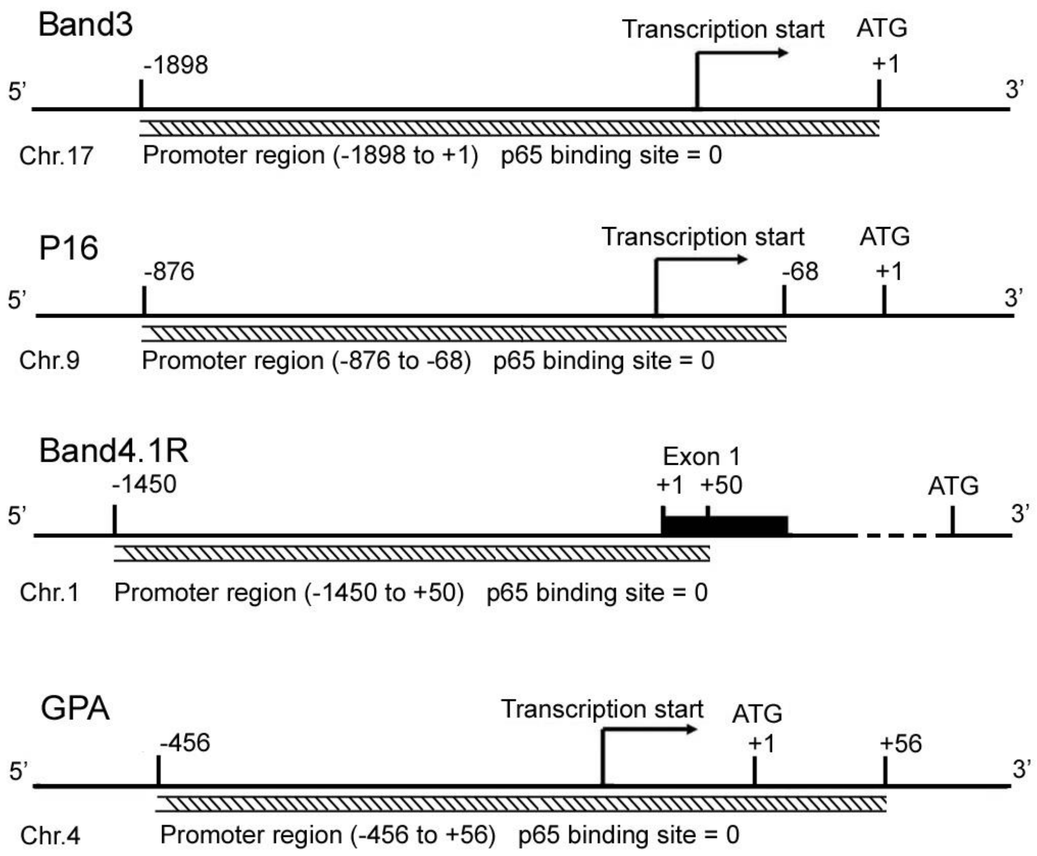

S2: Bioinformatics analysis showed that the entire promoter regions for band3, P16, GPA and band4.1R have no p65 binding sites

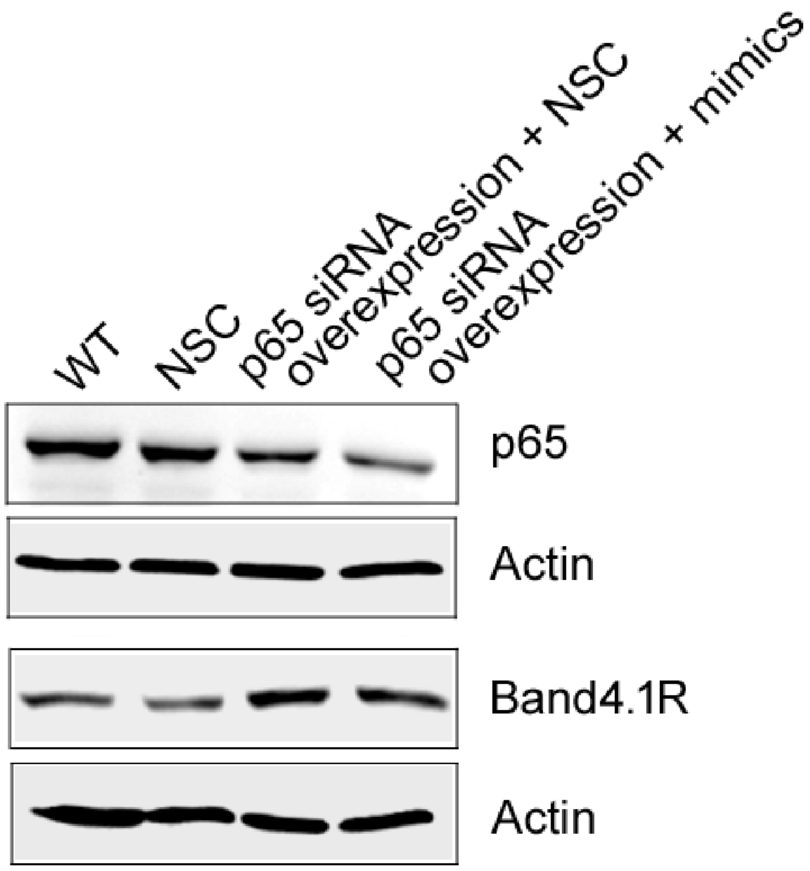

S3: Western blot analysis of band4.1R protein expression in K562 cells transfected with p65-targeted siRNA or p65-targeted siRNA plus miRNA mimics.

S4:. TargetScan 5.1 and RNA22 software were used to predict the binding sites for miR-23a, -27a and -24 in 3`UTR and CDS of 25 genes that are regulated during erythroid differentiation.

Target gene		Binding Sites											
		miR-23a				miR-27a				miR-24			
		CDS		3`UTR		CDS		3`UTR		CDS		3`UTR	
	Total	N	Sites	N	Sites	N	Sites	N	Sites	N	Sites	N	Sites
IGF-1R	17	2	3045~3065<br>3186~3206	3	6224~6244<br>7667~7687<br>8073~8093	4	559~579<br>1277~1297<br>1447~1467<br>2910~2930	3	4507~4527<br>7012~7032<br>8582~8602	1	2724~274<br>5	4	4548~4569<br>8527~8548<br>9011~9032<br>9322~9343
ADAMTS6	13			1	4668~4690	3	165~185<br>436~456<br>1113~1133	3	3507~3529<br>3604~3624<br>5596~5616	3	20~41<br>1127~1148<br>2673~269<br>4	3	3970~3992<br>5252~5273<br>5741~5762
ALK4	9			2	2430~2452<br>2558~2580	1	681~701	2	2560~2580<br>3354~3374			4	1963~1985<br>2508~2530<br>2816~2838<br>3609~3631
ADAM19	8			2	4141~4161<br>4524~4544	2	1279~1299<br>1568~1588	1	6209~6229	1	965~986	2	4346~4367<br>4570~4591
AE1/Band3	6					3	586~606<br>2285~2305			1	81~102	2	3297~3318<br>4245~4266

					2525~2545						
Band 4.1	5					1	3283~3305	2	1682~1702<br>1803~1823	2	3554~3574<br>4687~4708
SOCS6	5	2	2774~2796<br>4096~4118	1		1	1906~1928			1	4707~4729
IL-6R	4	1	2675~2695	1	813~832	1	1423~1443			1	2803~2824
Spectrin	4			2	12~32<br>403~423			2	1722~1743<br>7023~7044		
FOG-1	4							4	246~267<br>597~618<br>1389~1410<br>3060~3081		
GATA-2	4			1	877~897	1	1583~1605			2	1952~1973<br>2072~2093
Transferrin Receptor	4	1	2575~2597	2	87~107<br>1110~1130					1	4599~4620
NF-E2	3							3	314~335<br>336~357<br>767~788		
Bcl-XL	3							1	184~204	2	1403~1424

											1592~1613
SCL/TAL1	3					2	2580~2600<br>3440~3460	1	347~368		
Glycophorin A	3					2	1430~1449<br>2508~2530			1	1181~1202
GFI-1B	2			1	597~617					1	1195~1216
LMO2/RBTN2	2					1	756~776	1	209~230		
p16	2							1	347~368	1	862~883
FLT3	2			1	225~245			1	298~319		
ALA-Synthetase	1	1	270~290					0			
Hemoglobin, beta	1			1	29~49						
GATA-1	1							1	1049~1070		
EPOR	1							1	240~261		
IL-3R	1					1	1140~1160				
